# Rebranding asymptomatic type 1 diabetes: the case for autoimmune beta cell disorder as a pathological and diagnostic entity

**DOI:** 10.1007/s00125-016-4144-8

**Published:** 2016-10-26

**Authors:** Ezio Bonifacio, Chantal Mathieu, Gerald T. Nepom, Anette-G. Ziegler, Henry Anhalt, Michael J. Haller, Leonard C. Harrison, Matthias Hebrok, Jake A. Kushner, Jill M. Norris, Mark Peakman, Alvin C. Powers, John A. Todd, Mark A. Atkinson

**Affiliations:** 1 DFG Center for Regenerative Therapies Dresden, Faculty of Medicine, Technische Universität Dresden, Fetscherstrasse 105, 01307 Dresden, Germany; 2 Paul Langerhans Institute Dresden, German Center for Diabetes Research (DZD), Technische Universität Dresden, Dresden, Germany; 3 Clinical and Experimental Endocrinology, University Hospital of Leuven, Leuven, Belgium; 4 Benaroya Research Institute at Virginia Mason, The University of Washington School of Medicine, Seattle, WA USA; 5 Institute of Diabetes Research, Helmholtz Zentrum München, Neuherberg, Germany; 6 Forschergruppe Diabetes, Klinikum rechts der Isar, Technische Universität München, Neuherberg, Germany; 7 T1D Exchange, Boston, MA USA; 8 Department of Pediatrics, College of Medicine, University of Florida, Gainesville, FL USA; 9 Walter and Eliza Hall Institute of Medical Research, Melbourne, VIC Australia; 10 Department of Medical Biology, The University of Melbourne, Melbourne, VIC Australia; 11 University of California, San Francisco Diabetes Center, San Francisco, CA USA; 12 McNair Medical Institute, Pediatric Diabetes and Endocrinology, Baylor College Medical Center, Houston, TX USA; 13 Department of Epidemiology, Colorado School of Public Health, University of Colorado Anschutz Medical Campus, Aurora, CO USA; 14 Department of Immunobiology, King’s College London Faculty of Life Sciences & Medicine, London, UK; 15 National Institute of Health Research Biomedical Research Centre at Guy’s and St Thomas’ Hospitals and King’s College London, London, UK; 16 Division of Diabetes, Endocrinology, and Metabolism, Vanderbilt University School of Medicine, Nashville, TN USA; 17 VA Tennessee Valley Healthcare System, Nashville, TN USA; 18 JDRF/Wellcome Trust Diabetes and Inflammation Laboratory, Department of Medical Genetics, NIHR Cambridge Biomedical Research Centre, Cambridge Institute for Medical Research, University of Cambridge, Cambridge, UK; 19 Department of Pathology, Immunology, and Laboratory Medicine, College of Medicine, University of Florida, Gainesville, FL USA

**Keywords:** Autoimmunity, Beta cell, Diabetes, Disease classification

## Abstract

The asymptomatic phase of type 1 diabetes is recognised by the presence of beta cell autoantibodies in the absence of hyperglycaemia. We propose that an accurate description of this stage is provided by the name ‘Autoimmune Beta Cell Disorder’ (ABCD). Specifically, we suggest that this nomenclature and diagnosis will, in a proactive manner, shift the paradigm towards type 1 diabetes being first and foremost an immune-mediated disease and only later a metabolic disease, presaging more active therapeutic intervention in the asymptomatic stage of disease, before end-stage beta cell failure. Furthermore, we argue that accepting ABCD as a diagnosis will be critical in order to accelerate pharmaceutical, academic and public activities leading to clinical trials that could reverse beta cell autoimmunity and halt progression to symptomatic insulin-requiring type 1 diabetes. We recognize that there are both opportunities and challenges in the implementation of the ABCD concept but hope that the notion of ‘asymptomatic autoimmune disease’ as a disorder will be widely discussed and eventually accepted.

## A proposal for change

At what stage do we diagnose a disease? Do we require clinical signs and symptoms or is pathology sufficient? We present the case of asymptomatic beta cell autoimmunity in type 1 diabetes and argue for a conceptual shift in accepting this as a diagnostic entity.

Type 1 diabetes is a frequent chronic disease in childhood [1]. The disorder often presents acutely with metabolic decompensation that, in 30% of patients, includes a degree of ketoacidosis requiring immediate treatment and hospitalisation. A diagnosis of type 1 diabetes places significant psychological stress on the affected family, especially since upwards of 85% of new cases have no family history of the disease. Patients require life-long insulin therapy and clinical care because there are currently limited options for restoring insulin sufficiency and metabolic stability.

Here, we argue that type 1 diabetes should be diagnosed on the basis of pathology, primarily as an autoimmune disease, before it is diagnosed, classically, as a metabolic disease. The first signs of autoimmune pathology in type 1 diabetes are not clinically obvious, and pre-date the clinical metabolic presentation resulting from end-stage autoimmune beta cell destruction. By contrast, in many other autoimmune diseases, the clinical features and pathology are almost concomitant, such as the swollen, painful joints in rheumatoid arthritis. In these cases, upon the onset of symptoms, an active intervention treatment is promptly initiated. In this paradigm, early intervention means early in the course of disease and not during end-stage disease, i.e. when the joints in rheumatoid arthritis or the beta cells in type 1 diabetes have been destroyed.

A conceptual shift to a pathology-based diagnosis of type 1 diabetes has theoretical rigor and practical consequences. Making the diagnosis in the asymptomatic stage, prior to clinical presentation, has the potential to reduce the threat of ketoacidosis, alleviate psychological burden, allow for earlier initiation of experimental treatments that might preserve insulin sufficiency and reduce healthcare costs.

Our insights into the pathogenesis of type 1 diabetes and tools for detecting the onset of beta cell autoimmunity have evolved to the point where we can now identify individuals as ‘pre-clinical’, in whom beta cell autoimmunity will almost inevitably result in metabolic type 1 diabetes [2]. Autoantibodies against pancreatic beta cell antigens often develop in early childhood, precede the manifestation of hyperglycaemia in over 90% of patients, and are now diagnostic of future diabetes in children with or without a family history of type 1 diabetes [2, 3]. Therefore, we believe there is a strong case for recognising beta cell autoantibodies in asymptomatic children as a true diagnosis, reflective of a medical condition rather than just the predictive biomarker they are widely considered to represent. Moreover, while staging [3] serves the purpose to ‘date’ a disease process, we are advocating a shift in paradigm to type 1 diabetes being first and foremost an immune disease and only later a metabolic disease, presaging more active therapeutic intervention at the stage of autoimmunity, before clinical diagnosis.

We propose that a paradigm shift will be achieved through an accurate description of the autoantibody positive asymptomatic stage of type 1 diabetes, and that this description is provided by the name ‘autoimmune beta cell disorder’ (ABCD). This nomenclature was introduced to us by a highly informed patient with type 1 diabetes who remarked, ‘We need a name that indicates a pathogenic description of the stage, namely, overt autoimmunity, with a definite therapeutic objective, namely, to control or reverse autoimmunity’. We suggest that ABCD correctly reflects a disease that is abnormal and acquired, and if left untreated leads to life-threatening metabolic decompensation that requires life-long insulin therapy.

## Potential arguments to retain the status quo vs the benefits of change

A new diagnosis brings with it both opportunities and challenges. The physician must become acquainted with the new disorder. Both the physician and the patient would require education with regards to the benefits and risks of diagnosis, and the guidelines for diagnosis, monitoring and clinical care. Models for ABCD diagnosis and clinical care have been established in clinical research settings [3] and must be adapted for more widespread implementation. Opponents to widespread diagnostic application of ABCD may argue that its introduction does not yet provide a favourable cost:benefit ratio to the individual or society. While there are clear reductions in the rates of ketoacidosis and metabolic instability at diabetes onset in children with ABCD under clinical care [4], the screening and monitoring costs currently outweigh the savings in hospital care costs [5]. Currently, research funding largely covers the costs of ABCD diagnosis and care [3, 6]. This must transition to sustainable investment from non-research entities. An important step in this transition will be the assignment of a proper International Statistical Classification of Diseases and Related Health Problems (ICD) code [7] for ABCD, the direct consequence of which should be institutional remuneration for diagnosis and care. The importance of having a code that reflects the autoimmune pathology is underscored by the fact that the type 1 diabetes code is currently classified under endocrine, nutritional and metabolic diseases.

Opponents [8] also routinely raise concern that around half of children with ABCD will remain asymptomatic for months to years and, rarely, decades, before requiring treatment for dysglycaemia [2]. Therefore, they express concern that the lifestyle of a child may be adversely affected by the diagnosis of a condition potentially years before the appearance of symptoms. Their concern is supported by the fact that there are currently no treatments known to reverse ABCD that are, without question, safe enough to administer to asymptomatic children. However, in contrast to such opposing views, we believe that this current inability to reverse ABCD is a crucial argument for, and benefit of, introducing a formal diagnosis and name that recognises this condition as a pathology. Assigning a ‘disorder’ and ‘name’ to the pre-symptomatic autoimmune stage of type 1 diabetes will fundamentally change the perception and scope of therapeutics, from the treatment of type 1 diabetes and hyperglycaemia to the treatment and/or prevention of ABCD.

Beyond this, colleagues opposing our notion continue to suggest that diagnosis should wait until ABCD is manifest by metabolic signs and, therefore, close to end-stage beta cell failure [8]. In our opinion, this option would (by but one example) be analogous to waiting until joints are destroyed before attempting to treat rheumatoid arthritis. Moreover, it is currently not feasible to screen the population metabolically without first testing for beta cell autoantibodies and, as noted by Knip and colleagues [8], the sensitivity of metabolic testing for predicting clinical diabetes is still low. We are neither adverse to including metabolic testing nor to refining it. However, our ABCD concept calls for early diagnosis on the basis of antibodies that, when found to be multiple and are properly defined by biochemical assays, rarely revert [2, 9], so that treatment to reverse the autoimmune pathology becomes the objective. We find it concerning that to date, there have only been four appropriately powered randomised controlled trials of individuals with ABCD; this does not match our ability to diagnose ABCD. Even more concerning is that industry participation in these and the currently ongoing trials is limited to providing the study drugs. Moreover, if we listen to critics, the field is expected to learn from trials that are measured by metabolic outcomes and performed in adults with established metabolic disease in order to learn how to reverse autoimmunity or ABCD in children. In contrast, we argue that branding ABCD as a diagnosis will be a critical step in accelerating pharmaceutical, academic and public activities in therapeutic development and clinical trials, and therapies that effectively reverse beta cell autoimmunity and halt progression to symptomatic insulin-requiring type 1 diabetes.

Finally, introducing a diagnosis of ABCD will provide new opportunities for paradigm-shifting research. Assuming that health insurance providers, national medical care providers and the pharmaceutical industry begin to cover costs associated with the diagnosis and care of ABCD (and with clinical trials) we could expect that research funds currently used for these purposes could be spared and re-allocated to address more basic research questions. In the context of prevention, these include the identification of markers that identify treatment efficacy and markers that guide choice of treatment, as well as the identification of new therapeutic targets, improvement of existing candidate therapies and finding safe ways to combine therapies.

## The path forward

We believe that the concept represented by ABCD is scientifically sound and poised for clinical translation. It is likely to be the only way forward for the prevention of type 1 diabetes. We also suggest that the concept is not restricted to type 1 diabetes. Rather, it could be applied to, and transform, other childhood diseases where there are also likely benefits of pre-symptomatic diagnosis. We recognise that there are both opportunities and challenges to the implementation of such a concept, but hope that with the move towards precision medicine the notion of ‘asymptomatic autoimmune disease’ as a disorder will be widely discussed and eventually accepted. Indeed, academic, industrial, and public initiatives that are interested in applying precision medicine to children should consider this challenge.

## Abbreviation

ABCD: Autoimmune Beta Cell Disorder
